# Research Progress on Graphitic Carbon Nitride/Metal Oxide Composites: Synthesis and Photocatalytic Applications

**DOI:** 10.3390/ijms232112979

**Published:** 2022-10-26

**Authors:** Hao Lin, Yao Xiao, Aixia Geng, Huiting Bi, Xiao Xu, Xuelian Xu, Junjiang Zhu

**Affiliations:** Hubei Key Laboratory of Biomass Fibers and Eco-Dyeing & Finishing, College of Chemistry and Chemical Engineering, Wuhan Textile University, Wuhan 430200, China

**Keywords:** graphitic carbon nitride, metal oxides, heterojunctions, synthesis, photocatalytic applications

## Abstract

Although graphitic carbon nitride (g-C_3_N_4_) has been reported for several decades, it is still an active material at the present time owing to its amazing properties exhibited in many applications, including photocatalysis. With the rapid development of characterization techniques, in-depth exploration has been conducted to reveal and utilize the natural properties of g-C_3_N_4_ through modifications. Among these, the assembly of g-C_3_N_4_ with metal oxides is an effective strategy which can not only improve electron–hole separation efficiency by forming a polymer–inorganic heterojunction, but also compensate for the redox capabilities of g-C_3_N_4_ owing to the varied oxidation states of metal ions, enhancing its photocatalytic performance. Herein, we summarized the research progress on the synthesis of g-C_3_N_4_ and its coupling with single- or multiple-metal oxides, and its photocatalytic applications in energy production and environmental protection, including the splitting of water to hydrogen, the reduction of CO_2_ to valuable fuels, the degradation of organic pollutants and the disinfection of bacteria. At the end, challenges and prospects in the synthesis and photocatalytic application of g-C_3_N_4_-based composites are proposed and an outlook is given.

## 1. Introduction

With the development of economies and the growth of populations, pressures on energy demand and environmental pollution continue to increase all over the world [1,2,3,4]. Fossil fuels, which currently account for a large amount of the world’s energy, are increasingly consumed, resulting in negative impacts on the environment through the release of CO_2_, which is a serious greenhouse gas. Solar-energy-based photocatalysis is a promising technology to solve energy and environment problems, and has received extensive attention recently [5,6,7]. The synthesis of efficient photocatalysts is a key factor in applying photocatalytic technology to solve energy and environmental issues, such as water splitting to produce H_2_ and O_2_ [8,9,10,11,12], tail gas treatment (NO, CO_2_, etc.) [13,14], pollutant degradation [15,16,17,18,19,20], etc.

In the photocatalytic process, the electrons of photocatalysts are activated by absorbing photon energy [21]. Once the electrons have received enough energy, they will be excited to the valence band (VB), leaving holes at the conduction band (CB). The photogenerated electron–hole pairs (e^−^/h^+^) will then activate the reactants and promote the proceeding of a reaction [22,23]. In 1972, Fujishima and Honda reported the use of TiO_2_ electrodes for photocatalytic water splitting under ultraviolet light, which can be regarded as the milestone of photocatalytic technology [24]. In 1979, Inoue reported the reduction of CO_2_ into organic compounds in aqueous solution using TiO_2_, ZnO, GaP and CdS semiconductors [25]. Since then, the development of efficient semiconductors for photocatalysis has become a hotspot. Traditional photocatalysts mainly contained inorganic compounds, including metal oxides [26,27], sulfides [28], nitrides [29] and their composites [30], etc. The direct use of such materials was often restricted by their large band gap, which leads to low utilization efficiency for solar energy. Recently, graphite carbon nitride (g-C_3_N_4_) semiconductors have come into people’s horizons and have become a research focus in the field of photocatalysis, owing to their abundance, simple synthesis, high visible-light utilization efficiency and excellent physicochemical stability.

The application of g-C_3_N_4_ to photocatalysis was reported in 2009 by Wang et al. [31]. This material received widespread attention in photocatalysis thereafter owing to its polymeric properties and good visible-light response [32,33]. The challenge of applying g-C_3_N_4_ to photocatalysis mainly lies in its small specific surface area, narrow light response range and high e^−^/h^+^ recombination rate. To this end, many strategies have been proposed in the literature, such as adjustment of the microstructure [34,35,36], the doping of heteroatoms [37,38,39], the coupling of semiconductors [11,30,31,40,41,42], etc. Among these, coupling with other semiconductors is an attractive strategy, which can not only compensate for the shortcomings of g-C_3_N_4_ with their own properties, but also produce synergistic effects by forming heterojunctions. Both metal-free polymeric materials [43,44,45] and metal-containing inorganic materials, such as CdS [46], Fe_2_O_3_ [47] Fe_3_O_4_ [48], ZnO [49], TiO_2_ [50], Bi_2_WO_6_ [51] and Ce_2_(WO_4_)_3_ [52], can couple with g-C_3_N_4_ and form heterojunctions. In particular, the coupling of materials with special properties can give the composites interesting advantages. For example, the coupling of magnetic materials, e.g., g-C_3_N_4_/Fe_3_O_4_ [53] and g-C_3_N_4_/CoFe_2_O_4_ [54], can facilitate the recycling of photocatalysts (as they can be simply separated by a magnet), in addition to the improving photocatalytic performance.

**Figure 1:**
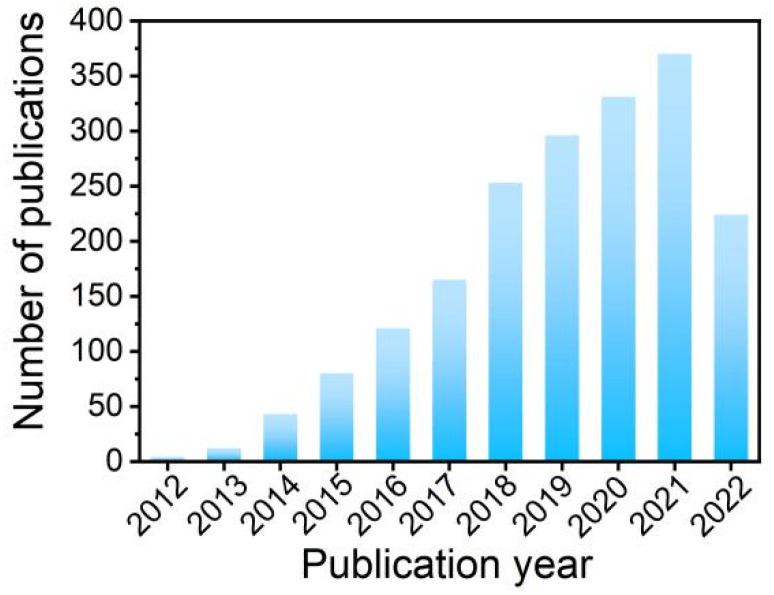
The number of publications on carbon nitride/metal oxide complexes for photocatalytic applications published in the last 10 years. Literature searched in Web of Science with the keywords: “carbon nitride” AND “metal oxide complex” AND “photocatalysis”.

Among the coupling materials, metal oxides came into the eyes of researchers early, because of their low-cost, abundance and easy synthesis. Many works on the coupling of g-C_3_N_4_ and metal oxides have been reported and great achievements have been made. The secular growth of related publications commendably reveals the flourishment of g-C_3_N_4_/metal oxide heterojunction materials in photocatalytic applications (Figure 1). To date, numerous breakthroughs and advances have been made in the photocatalysis system based on g-C_3_N_4_-based heterojunction materials, but a comprehensive summary still needs to be further subdivided, especially regarding the g-C_3_N_4_/metal oxide composite system. In this context, it is of great significance to summarize the recent advances in the synthesis and photocatalytic application of g-C_3_N_4_/metal oxide composites to alleviate environmental pollution and energy shortage. In detail, this work reviewed the recent progress on (1) the synthesis of g-C_3_N_4_ and its coupling with single or double metal oxides; (2) the photocatalytic applications of the composites in energy production and environmental protection; and (3) the challenges and prospects of g-C_3_N_4_-based heterojunction materials in photocatalytic applications. This review enables a wide range of researchers to understand these important areas and prospects, and the challenges and potential of g-C_3_N_4_/metal oxide composites.

## 2. Synthesis of g-C_3_N_4_ and Metal Oxides/g-C_3_N_4_ Composites

### 2.1. Synthesis of g-C_3_N_4_

With the in-depth study of g-C_3_N_4_ year-by-year, various modification strategies have been proposed and applied to improve the catalytic properties of g-C_3_N_4_ materials, including plasma sputtering deposition [55], solvothermal synthesis [56], chemical vapor deposition [57], thermal condensation [58], etc. The thermal condensation method receives special attention owning to its convenience, low-cost and time-savings. Nitrogen-rich materials, such as cyanamide [59], dicyandiamide [60], melamine [61], thiourea [62], urea [63], ammonium thiocyanate [64] and their mixtures [65], are generally used as the precursors to g-C_3_N_4_. However, this method often results in materials with low surface area and structural defects, which hinder the exposure of active sites on the surface [66] and act as the recombination centers of photogenerated electron–hole pairs, thereby reducing the photocatalytic performance. To solve these problems, it is suggested that the band gap structure of g-C_3_N_4_ be optimized to improve the separation efficiency of photogenerated e^−^/h^+^ pairs, and to adjust the microstructure to increase its specific surface area.

In this section, we mainly focus on the influence of precursors and preparation conditions on the properties of g-C_3_N_4_. In the case of precursors, cyanamide is first used to synthesize g-C_3_N_4_. In 2005, Antonietti et al. [67] prepared g-C_3_N_4_ via the thermal polymerization of cyanamide. In the process, cyanamide is first self-condensed to dicyandiamide at 150 °C, which then transforms to melamine, melem and, finally, g-C_3_N_4_ at 240 °C, 390 °C and 520 °C, respectively, accompanying the release of NH_3_. However, the high price, high toxicity and special transportation limit its wide use. Therefore, intermediate products with low cost, low toxicity and chemical stability, e.g., dicyandiamide and melamine, are generally used instead of cyanamide.

Ge et al. [68] used melamine as precursor to producing g-C_3_N_4_ at a temperature of 500~600 °C. They found that samples prepared at 520 °C showed the best performance for the photodegradation of phenol. This indicates that the properties of g-C_3_N_4_ depend intimately on the synthesis temperature, which promotes the modification of samples in, for example, the degree of crystallinity. Additionally, they also found that an increase in temperature can introduce nanostructures to the material, due to the exfoliation caused by the high temperature. This provides a way to control the structure and surface area of g-C_3_N_4_ with secondary thermal treatment, as is widely reported in the literature [69,70].

In addition to cyanamide and its derivates, other nitrogen-containing organics can also be used as precursors to g-C_3_N_4_. For example, Schaber et al. found that the thermal decomposition of urea in an open reaction vessel can yield g-C_3_N_4_, through the transformation of biuret, cyanuric acid, ammelide, ammeline and melamine intermediates [71]. Later, Liu et al. used urea as precursor to producing g-C_3_N_4_ without adding auxiliary agents, finding that the obtained material can show excellent activity for the photocatalytic degradation of methylene blue (MB) [72].

**Figure 2:**
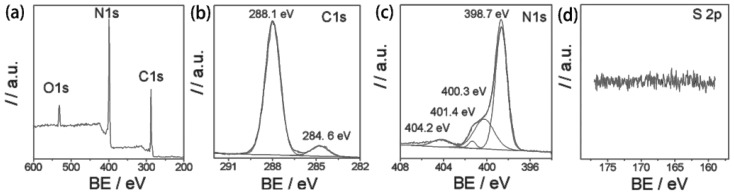
(**a**) XPS survey spectrum and the corresponding high-resolution spectra of (**b**) C ls, (**c**) N 1s and (**d**) S 2p obtained from the CN-T_500_ sample. Used with permission from [73]. Copyright 2012 Royal Society of Chemistry.

Zhang et al. investigated the reaction mechanism of transforming urea to produce a g-C_3_N_4_ network at high temperature, and found that the oxygen-containing groups of urea promote the condensation process [73]. In addition to urea, thiourea is also employed to fabricate g-C_3_N_4_, and it was found that the sulfur existing in thiourea changes the traditional monomer condensation pathway and plays a crucial role in optimizing the structure. In particular, no signal of sulfur was detected in the X-ray Photoelectron Spectroscopy (XPS) spectrum (Figure 2), which suggests that the sulfur acts as a medium rather than a component of the final material. In addition to the types of precursor, the polymerization temperature also affects the formation process of g-C_3_N_4_. The same authors found that the condensation of thiourea to g-C_3_N_4_ is insufficient at 450 °C, but could be completed at 500 °C. When the temperature continues increasing to 550 °C and 600 °C, the structure is optimized. However, the g-C_3_N_4_ starts to thermally decompose once the temperature is raised to 650 °C. One of the advantages of using thiourea as precursor is that it can induce the formation of nanostructured g-C_3_N_4_, as the oxygen in the structure gradually escapes at high temperatures. This further results in the exposure of surface sites and the localization of light-induced electrons in the conjugated systems, thereby improving the photocatalytic performance [74].

As mentioned above, urea serving as precursor can accelerate the production of large amounts of gases at high temperatures owing to the presence of oxygen in the structure, thereby improving the surface area of the product. For this reason, urea is often used as porogen in the preparation of g-C_3_N_4_ to increase the porosity, as well as the nitrogen content [75]. However, the excess addition of urea would produce a large number of fragments, which tend to agglomerate during the reaction, reducing the surface free energy and decreasing the photocatalytic activity. Therefore, the amount of urea added during synthesis is of great importance and worth being optimized.

For the improvement of surface area, Wu et al. [76] reported that the addition of NH_4_Cl additives during the synthesis procedure is also highly efficient, as they can be decomposed into HCl and NH_3_ gases during the heat-treatment process, promoting the delamination and depolymerization of g-C_3_N_4_, and thus, improving the surface area. Moreover, the presence of NH_4_Cl can lower the temperature of g-C_3_N_4_ formation to 400 °C and introduce numerous surface amino groups, which are beneficial to, for example, the photocatalytic H_2_ evolution reaction, with a reaction rate twice that of bulk g-C_3_N_4_. Similar cooperative effects are also observed for other multi-component systems, e.g., urea-mixed imidazole [77], or melamine and urea mixed with thiourea [78].

**Figure 3:**
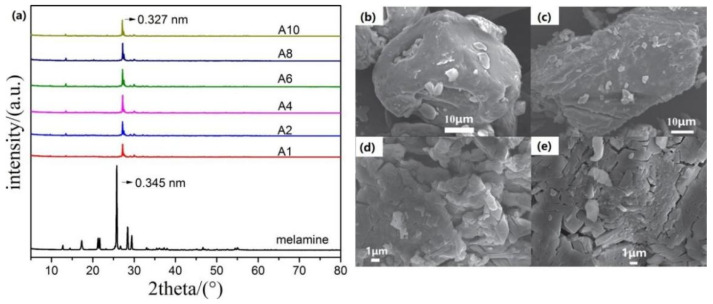
(**a**) XRD patterns of melamine and acid-treated melamine samples; SEM images of g-C_3_N_4_ prepared with (**b**) melamine and acid-treated melamine for (**c**) 2 h, (**d**) 6 h and (**e**) 8 h. Used with permission from [79]. Copyright 2019 IOP Publishing.

Pretreatment of the precursor is also an effective way to improve the surface area of g-C_3_N_4_. Sun et al. [79] prepared protonated g-C_3_N_4_ using HCl-treated melamine as a precursor and compared the effects of treatment time on the properties of the material. They found that the reaction of melamine with HCl changes the crystal structure and vibration bands of g-C_3_N_4_. Compared with g-C_3_N_4_ originating from untreated melamine, the material obtained from HCl-treated melamine exhibits smaller grain size and a bigger surface area. Powder X-ray diffraction (XRD) patterns shows that pretreatment with acid changes the structure of melamine, and shortens the formation process to 1 h (Figure 3a). The structure of the samples after treatment is similar, except for a slight shift in peak position due to the formation of nanosheets in the samples, which facilitates the strengthening of stacking between layers and reduction in the spacing distance [80]. Indeed, scanning electron microscope (SEM) images show that g-C_3_N_4_ obtained from the untreated melamine exhibited a particle size of 7.5 μm, which is larger than that obtained from HCl-treated melamine (Figure 3b). This verifies that acid-treated melamine prevents the thermal condensation of melamine into large-sized g-C_3_N_4_, by releasing HCl and NH_3_ gases in the heating process. Similar results are also reported for nitric acid-treated melamine [81] and sulfuric acid-treated melamine [82]. These results suggest that acid treatment of the precursor is beneficial to improve the surface area of g-C_3_N_4_, by generating cracks during the heating process. Moreover, the samples obtained from the acid-treated precursor possess the advantages of rich surface defects, excellent electron–hole separation efficiency and strong light absorption ability.

In addition to the acid pretreatment, the hydrothermal treatment of dicyandiamide also yields g-C_3_N_4_ with a high surface area and various surface morphologies, e.g., flower-like [83,84,85], hollow spheres [86,87,88], needle-like and rod-like [89,90,91], depending on the solvents. Such materials exhibit more attracting properties than the bulk one, for example: (1) the lamellar and porous structure is conducive to gas permeation; (2) the large surface area facilitates the reactant’s adsorption; (3) the special morphology provides the benefit of widening the visible-light response range and improving the light absorption ability.

**Figure 4:**
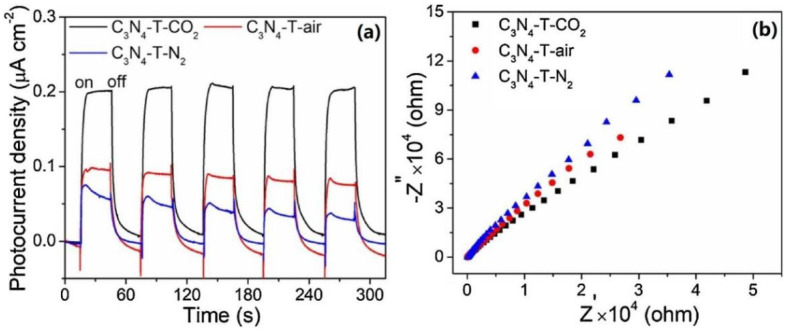
(**a**) Photocurrent responses and (**b**) EIS Nyquist plots of C_3_N_4_-T-Y under light irradiation. Used with permission from [94]. Copyright 2019 Elsevier.

As well as the precursor and temperature, the reaction atmosphere is also crucial in affecting the properties of g-C_3_N_4_, through generating carbon and nitrogen vacancies, for example. Wang et al. [66] fabricated nanorod g-C_3_N_4_/metal oxide composites by heating a copper–melamine supramolecular framework, [Cu(μ-OAc)(μ-OCH_3_)(MA)](Cu-MA1), under an argon atmosphere, which shows 94% Rhodamine B (RhB) conversion within 20 min under visible-light irradiation. Niu et al. [92] reported the generation of nitrogen vacancies by heating g-C_3_N_4_ in a hydrogen environment and foresaw the importance of self-modification and vacancies to completely modify the electronic structure of the layered g-C_3_N_4_ structure. Liang et al. [93] prepared porous g-C_3_N_4_ with abundant carbon vacancies by heating bulk g-C_3_N_4_ in a NH_3_ atmosphere. The obtained material showed a surface area of 196 m^2^/g, and exposed additional active edges, which significantly accelerated the transfer of photoinduced electron–hole pairs through a cross-plane diffusion pathway. The in-plane pores and wrinkled structures of g-C_3_N_4_ greatly enhance mass transfer and promote the dynamics of photoactivity. Xu et al. [94] reported that g-C_3_N_4_ prepared by pyrolyzing 3-amino-1,2,4-triazole in a CO_2_ atmosphere shows excellent activity for hydrogen production, which was 2.4 and 1.7 times higher than that prepared in air and N_2_ atmospheres, respectively. This could be because treatment in a CO_2_ atmosphere causes a reduction in nitrogen vacancies (V_n_) and the formation of NH_x_ groups on the surface of g-C_3_N_4_, generating hydrogen bond interactions between the layers, which facilitate the transfer of electrons from the heptazine ring to the g-C_3_N_4_ layer. Transient photocurrent response measurement confirms that the g-C_3_N_4_ prepared in a CO_2_ atmosphere produces the largest current density under visible-light driving (Figure 4a), which implies improvement in the separation efficiency of electron–hole pairs. Additionally, electrochemical impedance spectroscopy (EIS) shows that this sample has a smaller EIS arc radius than the others (Figure 4b) which confirms again that the g-C_3_N_4_ prepared in a CO_2_ atmosphere has lower charge transfer resistance and higher charge transfer efficiency.

Table 1 summarizes the band gap and surface area of g-C_3_N_4_ prepared under different reaction conditions, which shows that the selection of precursors and the proper control of reaction conditions are effective strategies to optimize the electronic structure and surface area of g-C_3_N_4_.

### 2.2. Synthesis of Single-Metal Oxide/g-C_3_N_4_ Heterojunctions

It is known that a single semiconductor often encounters problems such as low quantum yields, a narrow light absorption spectrum and low e^−^/h^+^ separation efficiency in photocatalysis, due to the contradiction between light absorption capability and e^−^/h^+^ recombination rate. Thus, modifications such as heteroatom doping, morphological control and semiconductor combination are often adopted in order to fully exhibit the photocatalytic properties of semiconductors. Metal oxides are one class of semiconductor and their inorganic characteristics can largely compensate for the shortcomings of polymeric g-C_3_N_4_. For example, the good redox ability of metal oxides can compensate for that of g-C_3_N_4_ when conducting redox reactions. Therefore, it would be of great interest to combine metal oxides with g-C_3_N_4_ to form inorganic–polymeric heterojunctions, which produce synergistic effects not only in the band gaps, but also in redox and other properties.

**Figure 5:**
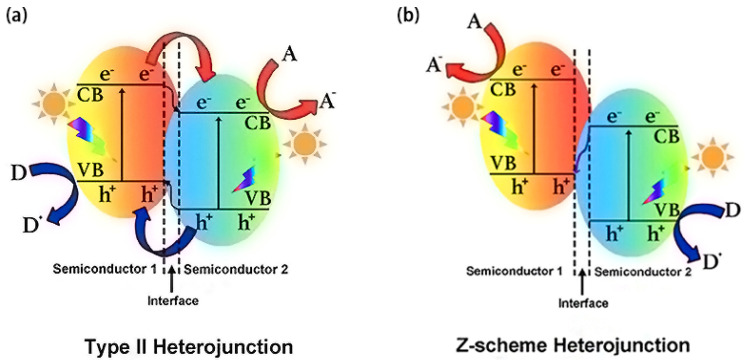
Band structure of (**a**) type II and (**b**) Z−scheme heterojunctions. “A” and “D” represent electron acceptor and electron donor, respectively. Used with permission from [100]. Copyright 2016 American Chemical Society.

The construction of heterojunctions requires unequal band levels between the semiconductors to create interface band arrangement, which can result in a built-in electric field to drive the opposite migration of photogenerated electrons and holes, improving e^−^/h^+^ separation efficiency. Type II and Z-scheme are two typical heterojunctions and their formation mechanisms are shown in Figure 5. The former requires two coupled semiconductors with an interleaved band structure. Thereby, the photo-generated electrons transfer from semiconductor 1 to semiconductor 2, and the holes transfer in the opposite direction (Figure 5a). The electrons accumulated on semiconductor 2 are used for a reduction reaction and the holes accumulated on semiconductor 1 are used for an oxidation reaction. This process can separate photo-generated electrons and holes in space, but sacrifices the redox capacity of the materials. Hence, both the oxidation potential and the reduction potential of the heterojunction are reduced compared to those of the semiconductor alone.

A Z−scheme heterojunction requires the components to have staggered energy-band configurations, with the electron transfer in a zigzag mode. Typically, the photogenerated electrons at the CB of semiconductor 2 transfer and combine with the holes at the VB of semiconductor 1. The retained electrons at the CB of semiconductor 1 and holes at the VB of semiconductor 2 participate in the reduction and oxidation reactions, respectively (Figure 5b). This charge transfer mode enables the system to have not only improved charge-separation efficiency, but also stronger redox capability compared to that of the sole semiconductor.

Among the metal oxides, TiO_2_ is well known for its first application to photocatalysis. Generally, it has three polymorphs in nature, including anatase, rutile and brookite [101]. Rutile TiO_2_, with a band gap of 3.0 eV, has the most stable and compact structure, while anatase TiO_2_, with a band gap of 3.2 eV, is better facilitates photocatalysis owing to its good e^−^/h^+^ separation efficiency and high adsorption capacity [102]. In the fabrication of TiO_2_ and g-C_3_N_4_ heterojunctions, Fang et al. [103] prepared an anatase/rutile TiO_2_/g-C_3_N_4_ (A/R/CN) multi-heterostructure using a facile thermoset hybrid method, finding that the combination of two type II heterostructures (i.e., A/R and R/CN) greatly improved the separation and transfer efficiency of e^−^/h^+^. As a result, the heterostructures showed activity that was eight and four times higher for the photocatalytic hydrolysis of hydrogen than g-C_3_N_4_ and TiO_2_ (P25) alone, respectively. Similarly, other TiO_2_ polymorphs, e.g., brookite TiO_2_, can combine with g-C_3_N_4_ to prepare heterojunctions with improved photocatalytic activity [104]. Zhu et al. prepared g-C_3_N_4_/TiO_2_ hybrids via a ball-milling method, finding that the composites possess a wider light-absorption range and higher photocatalytic activity than the respective component, with the activity for MB degradation being 3.0 and 1.3 times higher than that of g-C_3_N_4_ and TiO_2_, respectively [105].

In addition to TiO_2_, the combination of g-C_3_N_4_ with other metal oxides is also widely reported. Liu et al. reported that the coupling of g-C_3_N_4_ with ZnO prolongs the lifetime and separation efficiency of photogenerated e^−^/h^+^, therefore improving its photocatalytic activity for phenol degradation [106]. Moreover, the introduction of a silicate group to the ZnO/g-C_3_N_4_ composites further improves the lifetime and separation efficiency of e^−^/h^+^ pairs, and thereby, the photocatalytic activity. This indicates that the built-in silicate group in the composites acts as a bridge to link ZnO and g-C_3_N_4_, promoting the transfer and separation efficiency of e^−^/h^+^ pairs. Consequently, the electrons and holes have a longer lifetime to interact with the reactants and contribute to the reaction.

**Figure 6:**
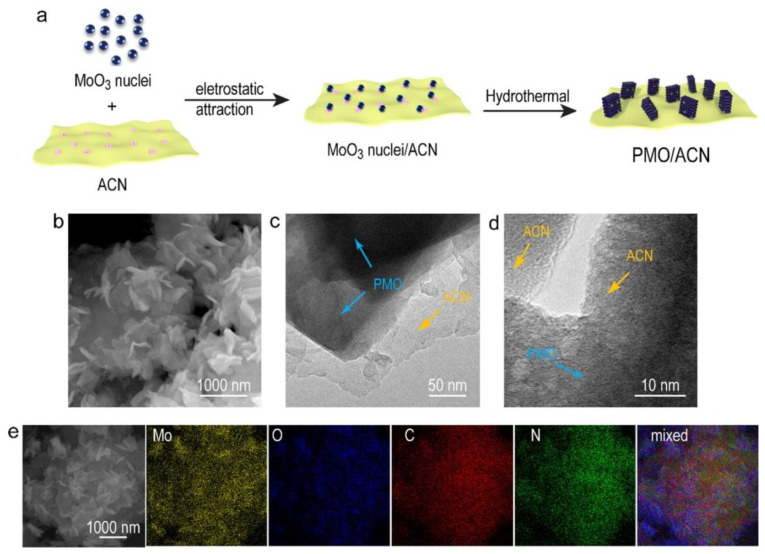
Synthesis of the PMO/ACN nanohybrid. (**a**) Schematic illustration for the synthesis of PMO/ACN, (**b**) SEM, (**c**) TEM, (**d**) HRTEM images of the PMO/ACN sample and (**e**) SEM elemental mapping for the PMO/ACN nanohybrid. Used with permission from [107]. Copyright 2020 Elsevier.

Guo et al. [107] coupled oxygen-deficient molybdenum oxide (MoO_3_) nanoplates with g-C_3_N_4_ nanoplates using a one-step hydrothermal method, and found that MoO_3_ particles grew well on the surface of g-C_3_N_4_ (Figure 6). MoO_3_ is a chemically inert semiconductor with a large work function, which is suitable to couple with g-C_3_N_4_ and form a Z-scheme heterojunction. Moreover, the oxygen vacancies facilitate the promotion of plasmon resonance and expand the range of spectral absorption, and their concentrations can be adjusted via annealing in air. Combined with surface plasmon resonance and the synergistic effects of Z-scheme heterojunctions, it is expected that the composites will exhibit efficient performance for photocatalytic reactions, e.g., the H_2_ evolution reaction.

**Figure 7:**
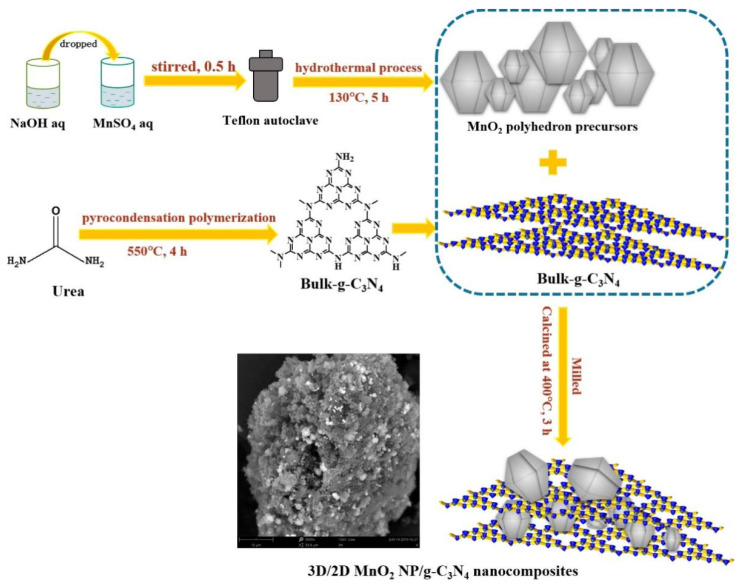
Synthesis procedures of 3D/2D MnO_2_ NP/g-C_3_N_4_ nanocomposites. Used with permission from [108]. Copyright 2019 American Chemical Society.

The morphology, structure and contacting patterns are also crucial factors affecting the electron transfer and photocatalytic activity of g-C_3_N_4_. Using seed-induced solvent heat treatment, 0D nanoparticles, 1D nanowires, 2D nanosheets and 3D mesoporous crystals can be loaded on the surface of g-C_3_N_4_. For example, 3D/2D MnO_2_/g-C_3_N_4_ nanocomposites can be prepared via a calcination process using MnO_2_ polyhedron and 2D g-C_3_N_4_ nanosheets as precursors [108], as shown in Figure 7. The 3D polyhedral morphology and multi-phase polycrystalline structure of MnO_2_ are beneficial as they strengthen the interaction between MnO_2_ and g-C_3_N_4_, owing to the presence of low-valence Mn species, graphitic N species and oxygen vacancy. Like the lamellar structure, materials with other structures can increase the interfacial area and surface area of the resulting composites. Liu et al. [109] reported that core-shell CeO@g-C_3_N_4_ exhibits high efficiency for the photocatalytic degradation of doxycycline, owing to the high surface area (82.37 m^2^/g) and low e^−^/h^+^ recombination rate. They also found that shuttle-like CeO_2_/g-C_3_N_4_ is efficient for the degradation of norfloxacin under visible light using persulfate as an oxidant, during which the norfloxacin is degraded into small molecules via gradual shedding of the functional groups.

The unique properties of metal oxides also give the composites special functions. Ye et al. [110] loaded magnetic Fe_2_O_3_ on g-C_3_N_4_ to introduce magnetization to the sample, which makes it easy to separate from the reaction liquid, and hence, reduces the cost of the recycling process. Mou et al. [111] used amorphous ZrO_2_ as a cocatalyst of g-C_3_N_4_ for ammonia synthesis to improve its activity. The introduction of ZrO_2_ not only restrains the hydrogen generation rate, but also improves the electron transfer rate and the e^−^/h^+^ separation efficiency. These results demonstrate that the combination with metal oxide is efficient in improving the photocatalytic performance of g-C_3_N_4_. Table 2 and Table 3 summarize the recent advances in metal oxide/g-C_3_N_4_ heterojunctions in photocatalytic applications.

### 2.3. Multiple-Metal Oxide/g-C_3_N_4_ Heterojunctions

The achievements in combining single-metal oxide with g-C_3_N_4_ have stimulated researchers to use multiple-metal oxides to upgrade the materials. The construction of multi-component composites can induce multi-step charge transfer and charge separation, and hence, better photocatalytic performance could be expected when compared to single ones. However, more attention should be paid to the matching of the energy-band potential of each component, so that the photo-generated electrons can transfer at the phase interface, reaching the goal of constructing heterojunctions.

**Figure 8:**
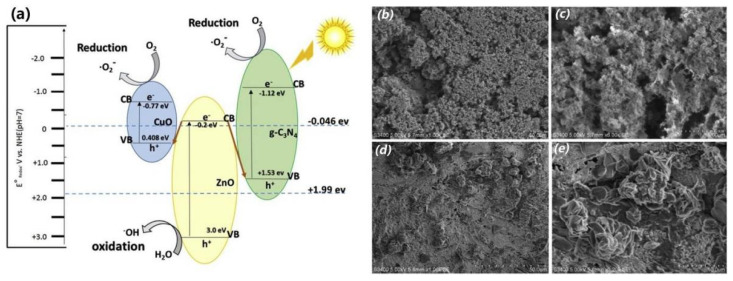
(**a**) Schematic diagram of the charge migration pathway in CuO/ZnO/g-C_3_N_4_; SEM images of (**b**,**c**) CuO/ZnO and (**d**,**e**) CuO/ZnO/g-C_3_N_4_ with different magnifications. Used with permission from [135]. Copyright 2019 Elsevier.

Bajiri et al. constructed ternary and double Z-scheme CuO/ZnO/g-C_3_N_4_ heterojunctions using a solvothermal method [135], which consisted of g-C_3_N_4_ flakes decorated with small nanoparticles (<5 nm) (Figure 8). It is interesting to find that the gases released from the solute combustion process build up a porous structure in the material, similar to the function of porogens. The porous and sheet-like structure increases the capability of the material to absorb reactants on the surface, thereby improving the photodegradation efficiency. Indeed, the material exhibits activity of 98% (45 min) and 91% (6 h) for the degradation of MB and ammonia nitrogen, respectively, under visible-light irradiation.

**Figure 9:**
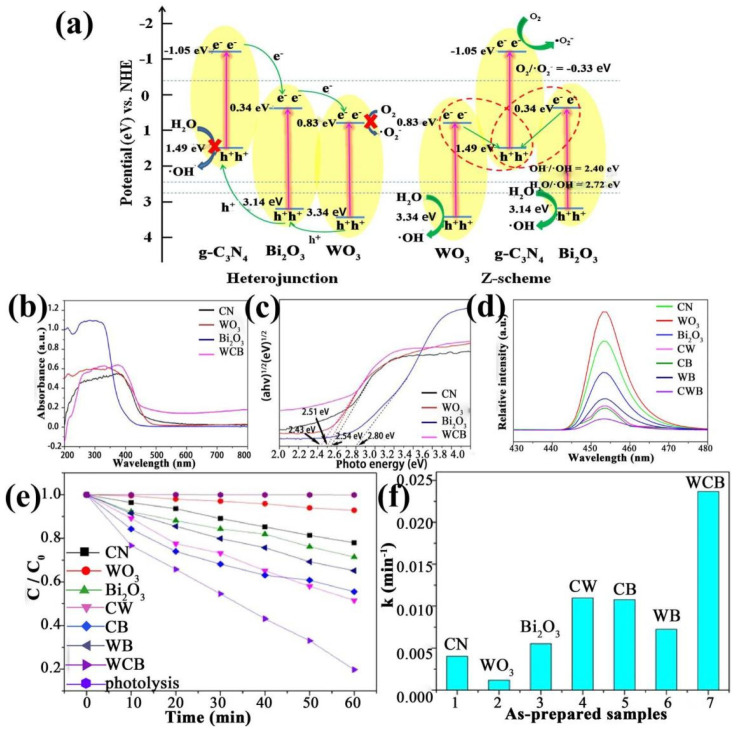
(**a**) Schematic diagram of charge separation in WO_3_/g-C_3_N_4_/Bi_2_O_3_, (**b**) UV-vis spectra, (**c**) band gap, (**d**) photoluminescence spectra, (**e**) photocatalytic activities for TC degradation under visible-light and (**f**) apparent rate constants for TC degradation obtained from the various samples. Used with permission from [136]. Copyright 2018 Elsevier.

Jiang et al. [136] found that in addition to acting as photocatalyst, g-C_3_N_4_ can be an intermediate for charge transfer, by constructing a WO_3_/g-C_3_N_4_/Bi_2_O_3_ (WCB) catalyst. Compared to the single or binary materials, ternary WCB exhibits moderate surface area and the highest photocatalytic activity. This indicates that the high surface area facilitated the reaction but was not the key factor determining the reaction. Optical characterizations from the UV-vis and PL spectra showed that the light absorption edge is red-shifted and the e^−^/h^+^ recombination rate is inhibited for WCB, when compared to the single or binary counterparts, due to the interactions between WO_3_, g-C_3_N_4_ and Bi_2_O_3_ (Figure 9a). Consequently, the WCB exhibits enhanced optical properties and improved photocatalytic activity for tetracycline (TC) degradation under visible-light irradiation, with TC conversion of 80.2% at 60 min, which is much higher than that of g-C_3_N_4_ (22.1%), WO_3_ (7.17%) and Bi_2_O_3_ (28.6%), and the binary CW (g-C_3_N_4_/WO_3_), CB (g-C_3_N_4_/Bi_2_O_3_) and WB (WO_3_/Bi_2_O_3_) (Figure 9b–f).

**Figure 10:**
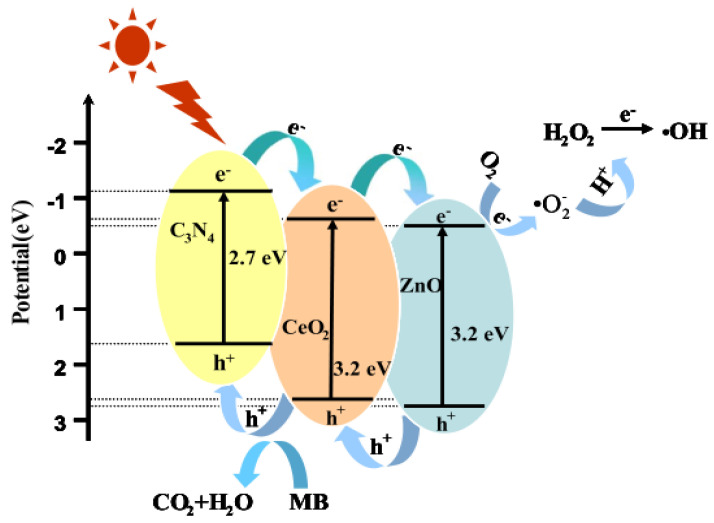
Schematic diagram of the photoexcited e^−^/h^+^ separation process in the g−C_3_N_4_/CeO_2_/ZnO composite under visible-light irradiation. Used with permission from [137]. Copyright 2017 Elsevier.

Yuan et al. [137] constructed ternary g-C_3_N_4_/CeO_2_/ZnO composites with multiple heterogeneous interfaces. Binary g-C_3_N_4_/CeO_2_ nanosheets were first prepared via pyrolysis and exfoliation. Thereafter, spherical ZnO nanoparticles were anchored on the g-C_3_N_4_/CeO_2_ surface to form a ternary heterojunction structure. Because of the formation of the type II staggered belt arrangement between the components, the g-C_3_N_4_/CeO_2_/ZnO shows efficient three-level transfer of electrons and holes, resulting in the effective separation of photo-excited carriers, as shown in Figure 10.

**Figure 11:**
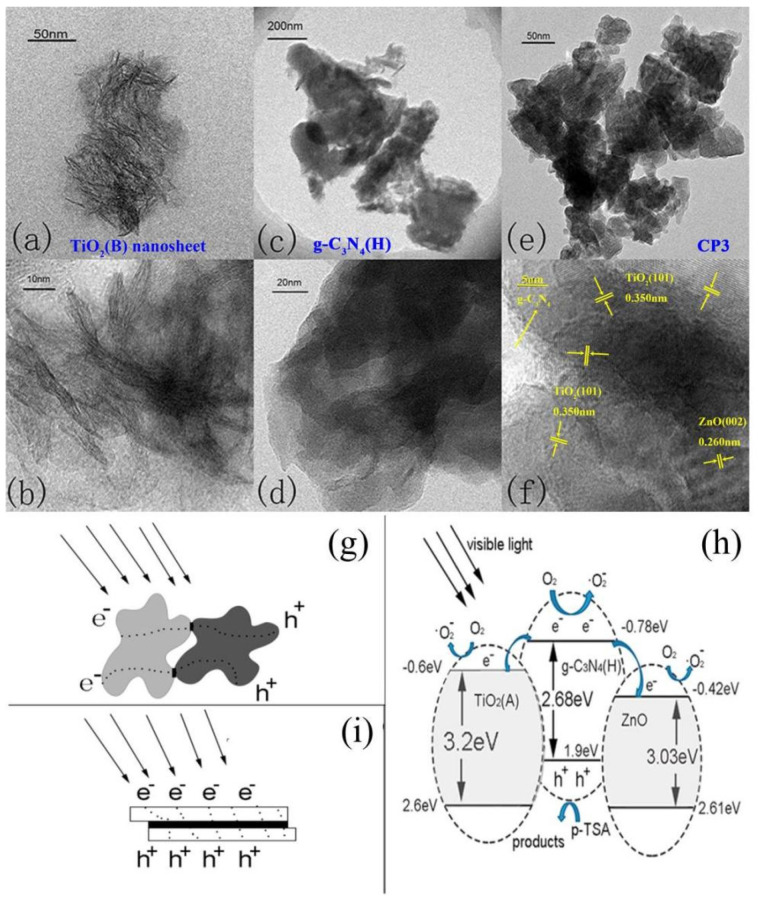
TEM images of (**a**,**b**) TiO_2_ nanosheets, (**c**,**d**) g-C_3_N_4_ nanosheets and (**e**,**f**) g-C_3_N_4_/TiO_2_/ZnO nanocomposites; (**g**,**i**) comparison of the electron transfer routes between the granule/granule and the nanosheet/nanosheet composites with different heterojunction areas; (**h**) the mechanism of facet-coupled ternary nanocomposites for p-TSA degradation under visible light. Used with permission from [138]. Copyright 2017 Elsevier.

Morphology control is also effective in improving the photocatalytic performance of materials, by enhancing the interactions and enlarging the contact areas of the heterogeneous interfaces, which are beneficial to electron transfer and separation (from the holes). For example, Jiang et al. [138] fabricated g-C_3_N_4_, TiO_2_ and ZnO nanoflakes, and then, assembled them to form g-C_3_N_4_/TiO_2_/ZnO Z-scheme heterojunctions. A high-resolution TEM image shows that the TiO_2_ (101) plane and ZnO (002) plane are stacked on the g-C_3_N_4_ surface to form heterojunctions (Figure 11a–f). The similar morphologies of g-C_3_N_4_/TiO_2_/ZnO and g-C_3_N_4_ indicate that TiO_2_ and ZnO are uniformly dispersed on the g-C_3_N_4_ surface. The 2D/2D nanosheet/nanosheet structure not only increases the surface area of the material (g-C_3_N_4_: 8.18 m^2^/g, g-C_3_N_4_/TiO_2_/ZnO: 27.21 m^2^/g), but also improves the e^−^/h^+^ separation efficiency by facilitating electron transfer through the abundant interfaces (Figure 11g–i).

**Figure 12:**
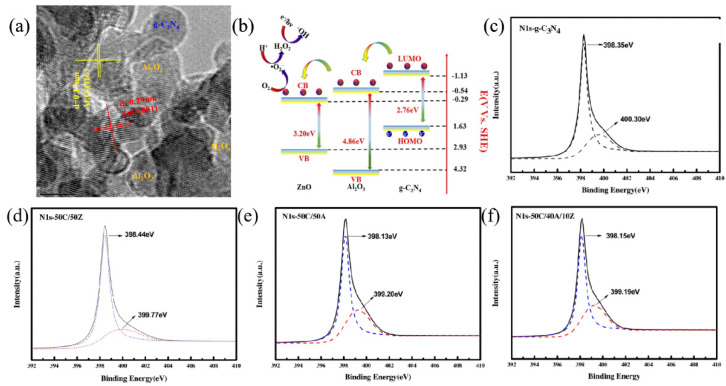
(**a**) HRTEM image of ternary g-C_3_N_4_/Al_2_O_3_/ZnO heterojunctions. (**b**) Cascade of electron transfer in ternary g-C_3_N_4_/Al_2_O_3_/ZnO heterojunctions under visible-light irradiation; high−resolution XPS spectra of (**c**) Al2p. (**d**–**f**) N1s of g-C_3_N_4_, 50C/50Z, 50C/50A and 50C/40A/10Z. Used with permission from [139]. Copyright 2017 Elsevier.

The formation mechanism of the composites has been explored to reveal how the heterogeneous interfaces affect the electron transfer process. Liu et al. [139] proposed a lattice-matching assumption of amorphous materials in the structural hybridization process and clarified a coordination effect in the unoccupied *d* orbitals of N atoms of g-C_3_N_4_. Because of the different crystal structures and lattice parameters of metal oxides (e.g., ZnO) and g-C_3_N_4_, lattice matching between them is difficult. Amorphous materials (e.g., Al_2_O_3_) have disordered atomic distribution and unfixed lattice parameters; hence, they can easily accept the charge of g-C_3_N_4_. Therefore, amorphous Al_2_O_3_ can be an intermediary to improving electron transfer efficiency between g-C_3_N_4_ and ZnO [140]. As shown in Figure 12a, the lattice fringes of ZnO and Al_2_O_3_ are entangled with that of g-C_3_N_4_, which proves that the two components are in close contact. The tight contact interface provides a step to transfer the induced carrier (Figure 12b). XPS spectra show that the binding energies of Al atoms in g-C_3_N_4_/Al_2_O_3_ and g-C_3_N_4_/Al_2_O_3_/ZnO shifted to a higher position compared to that of the original Al_2_O_3_ (Figure 12c). This indicates that a chemical force between Al and g-C_3_N_4_ is formed, due to the coordination of the unoccupied 3p or 3d orbital of Al ions with the lone electron pair of N atoms of g-C_3_N_4_, as verified by the shift in the binding energy of N 1s (Figure 12d–f). These results provide ideas to correct the lattice mismatch between g-C_3_N_4_ and metal oxides, and promote the application of amorphous materials to fabricate heterojunctions.

**Figure 13:**
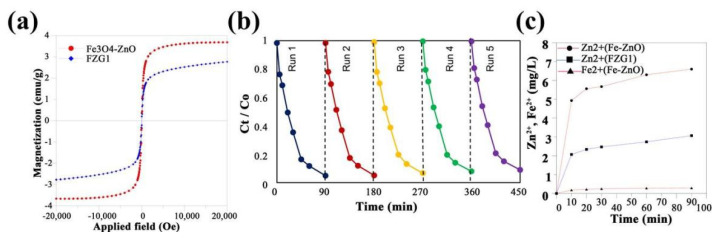
(**a**) Magnetization curves of Fe_3_O_4_-ZnO and FZG1; (**b**) release of zinc and iron ions into the solution as a function of time for Fe-ZnO and FZG1; (**c**) recyclability of FZG1 for photocatalytic degradation of SMX. Used with permission from [142]. Copyright 2018 Elsevier.

Fe_3_O_4_ is an attractive material in the synthesis of multi-component heterojunctions, owing to its good photocatalytic and especially magnetic properties, which promote not only the reaction activity but also the separation efficiency of catalysts from liquid solutions. Adil Raza et al. [141] prepared a Fe_3_O_4_/TiO_2_/g-C_3_N_4_ composite using a hydrothermal method, finding that anatase TiO_2_ and magnetic Fe_3_O_4_ can enter the g-C_3_N_4_ frame if treated at 200 °C. The composites show efficient activity for RhB and MO degradation under visible-light irradiation, with degradation conversions of 96.4% and 90%, respectively, which are 3.73 and 2.74 times higher than that of g-C_3_N_4_. Amir Mirzaei [142] prepared petal-like Fe_3_O_4_-ZnO@g-C_3_N_4_ composites using an in situ growth method, finding that the hydrolysis of urea (precursor) produces stable and continuous OH^−^ ions, which can react with zinc ions and control the growth of nuclei. The coating of g-C_3_N_4_ corrodes the surface of Fe_3_O_4_-ZnO and creates pores in the structure, benefiting electron transfer, while the presence of Fe_3_O_4_ not only reduces the e^−^/h^+^ recombination rate by accepting useless electrons, but also improves the separation efficiency of catalysts from solution (via a magnet) owing to its magnetic properties (Figure 13a). Moreover, the composite is stable and no leaching of Zn^2+^ and Fe^2+^ ions is observed in the reaction of photocatalytic SMX degradation (Figure 13b,c). This suggests that g-C_3_N_4_ acts not only as a semiconductor contributing to the photocatalytic reaction, but also a protective layer against photo-corrosion of the Fe-ZnO surface. Similar phenomena are observed for other composites, e.g., Ag_2_O/g-C_3_N_4_/Fe_3_O_4_ [53], ZnO/Fe_3_O_4_/g-C_3_N_4_ [143] and α-Fe_2_O_3_/g-C_3_N_4_/ZnO [144].

**Figure 14:**
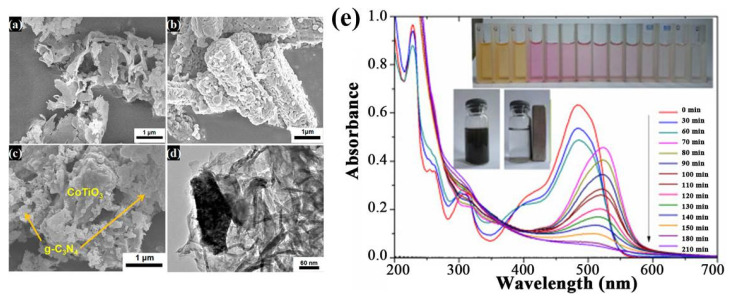
SEM images of (**a**) g-C_3_N_4_, (**b**) CoTiO_3,_ (**c**) CT-U and (**d**) 0.15% CT-U. Used with permission from [149]. Copyright 2016 American Chemical Society; (**e**) the photodegradation of Orange II by CuFe_2_O_4_@C_3_N_4_/H_2_O_2_/Vis system (inset: the solution before and after magnetic separation using an external magnet). Used with permission from [150]. Copyright 2015 Elsevier.

In addition to simple metal oxide, compound oxides are also interesting materials in catalysis, and they usually exhibit different electronic and chemical properties relative to their parent materials. Many compound metal oxides have been used to couple with g-C_3_N_4_ and form heterojunctions. Among them, perovskite oxides with an ABO_3_ structure attract much attention owing to their unique physical and chemical properties, such as variable ion valences, controllable oxygen vacancies, adjustable redox properties and the ability to accommodate foreign ions [145,146,147]. The typical perovskite oxide, CaTiO_3_ (CT), has a band gap of ~3.5 eV, which means that its photocatalytic activity is limited to ultraviolet excitation. However, when it is coupled with narrow-band-gap semiconductors such as g-C_3_N_4_ to form binary heterojunctions, the large band gap of CT can efficiently enhance the photocatalytic activity of g-C_3_N_4_ under visible light by promoting the charge-separation efficiency. Kumar et al. [148] found that the combination of 2D CT nanosheets with g-C_3_N_4_ flakes, to form 2D/2D composite nanoflake (CT/CN), greatly increased the BET surface area to 50.7 m^2^/g, which is larger than that of CT nanosheets (29.3 m^2^/g) and g-C_3_N_4_ flakes (41.0 m^2^/g). Hence, more active sites can be exposed on the surface, shortening the bulk diffusion length and reducing the e^−^/h^+^ recombination rate. Ye et al. [149] reported that the fabrication of 1D CoTiO_3_ rod–2D g-C_3_N_4_ flake Z-scheme heterojunctions (CT-U) improves not only e^−^/h^+^ separation efficiency, but also redox ability. SEM images show that the CoTiO_3_ rods are fully wrapped with g-C_3_N_4_, forming heterogeneous interfaces that are beneficial to electron transfer (Figure 14a–d). As a result, CT-U exhibited a reaction rate of 858 μmol/h/g for the hydrogen evolution reaction, which is about two times higher than that obtained from g-C_3_N_4_.

The effects of surface morphology on photocatalytic activity are documented by Zhang et al. [151], who used KNbO_3_ as a model catalyst and found that its efficiency for the photocatalytic conversion of methanol to hydrogen depends intimately on the morphology, with an order of cubic > orthogonal > tetragonal. On this basis, a cubic KNbO_3_/g-C_3_N_4_ composite was synthesized and it exhibited excellent activity for photocatalytic hydrogen production, owing to the close contact between KNbO_3_ cubes and g-C_3_N_4_ nanosheets, which forms active heterojunction interfaces and effectively inhibits the e^−^/h^+^ recombination rate in the system [152]. With the same principle, many ABO_3_/g-C_3_N_4_ composites are prepared and reported in the literature, such as LaFeO_3_/g-C_3_N_4_ [153], g-C_3_N_4_/SrTiO_3_ [154] and LaMnO_3_/g-C_3_N_4_ [155].

As well as perovskite oxides, spinel oxides with an AB_2_O_4_ structure are also promising materials in catalysis [156,157]. Compared to ABO_3_ perovskites, AB_2_O_4_ spinels have a narrower band gap and stronger responses to visible light. Moreover, the AB_2_O_4_ spinels can accommodate transitional metals at both the A- and B-sites; thus, the metals at both the A- and B-sites can contribute to the reactions. For example, Chang et al. [158] reported that Z-scheme NiCo_2_O_4_/g-C_3_N_4_ heterojunctions exhibit not only a larger surface area (141.7 m^2^/g) than g-C_3_N_4_ (89.2 m^2^/g) and NiCo_2_O_4_ (98.8 m^2^/g), but also higher photo-activity for water splitting than Co_3_O_4_/g-C_3_N_4_ and NiO/g-C_3_N_4_, owing to their abundant active sites and good photoelectric properties.

Some spinel oxides (e.g., CuFe_2_O_4_) also have magnetic properties, exhibiting the advantages of easy separation. In the research of Yao et al. [150], they constructed a type II CuFe_2_O_4_@g-C_3_N_4_ heterojunction, in which the CuFe_2_O_4_ and g-C_3_N_4_ are intertwined to form a three-dimensional hybrid structure that is beneficial to electron transfer. As a result, the material shows improved e^−^/h^+^ separation efficiency and photocatalytic activity compared to the respective g-C_3_N_4_, CuFe_2_O_4_ and g-C_3_N_4_/CuFe_2_O_4_ mixtures. Moreover, the material exhibits good easy-to-separate magnetism owing to its magnetic properties (Figure 14e), and thus, can be well recycled in the reaction.

## 3. Applications of g-C_3_N_4_-Based Photocatalysts

Its promising optical and physicochemical properties enable g-C_3_N_4_, utilizing sunlight, to solve the problems of environmental pollution and energy crises, while avoiding secondary pollution. In the following, we briefly introduce the application of g-C_3_N_4_-based materials in photocatalysis [159,160,161,162], including water splitting to generate H_2_ and O_2_, the degradation of pollutants, CO_2_ reduction and bacterial disinfection.

### 3.1. Photocatalytic Water Splitting for H_2_

**Figure 15:**
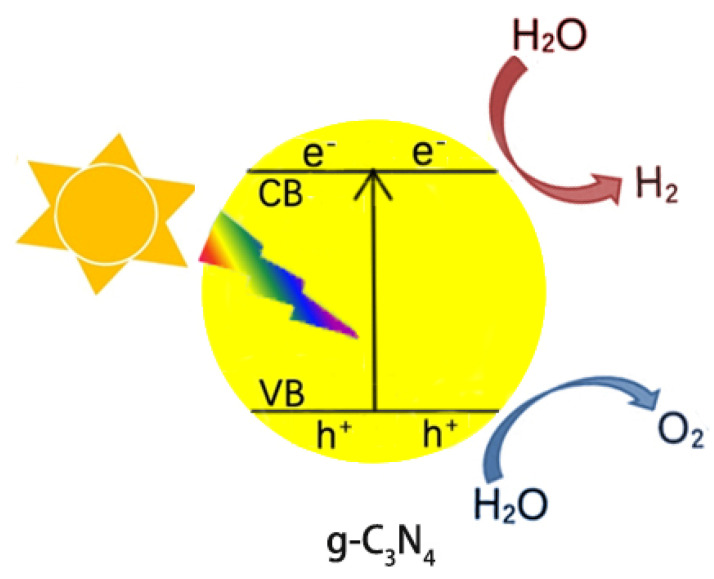
Scheme of photocatalytic water splitting into H_2_ and O_2_ over g-C_3_N_4_ under light irradiation.

Because of the decreasing storage of fossil fuels and their negative impacts on the environment (releasing CO_2_ for example), the use of green and renewable hydrogen fuels attracts much attention from scientists. The photocatalytic splitting of water is an ideal way to generate hydrogen and has become a hot topic in recent years. Figure 15 presents a simplified diagram of splitting water into hydrogen and oxygen over g-C_3_N_4_ under light irradiation. First, g-C_3_N_4_ is excited by photons to generate electrons, which then jump to the CB, leaving holes at the VB. The photogenerated e^−^ and h^+^ flow to the surface of g-C_3_N_4_, reducing and oxidizing the adsorbed water to hydrogen and oxygen, respectively. However, the generated e^−^/h^+^ will rapidly recombine each other due to the Coulombic attraction, losing activity. The improvement in the separation efficiency of the photogenerated e^−^/h^+^ pairs, thus, is a challenging topic in the field of g-C_3_N_4_ photocatalysis.

To achieve this, the coupling of g-C_3_N_4_ with metal oxide is a solution, which can separate e^−^/h^+^ pairs in space by forming an opposite flow of e^−^ and h^+^ (for type II heterojunctions), or by inducing the recombination of unused e^−^ and h^+^ (for Z-Scheme heterojunctions), as reported in the literature [163,164]. Shi et al. [165] reported the in situ synthesis of MoO_3_/g-C_3_N_4_, via co-pyrolysis of MoS_2_ and melamine, for photocatalytic water splitting to hydrogen, finding that the activity of g-C_3_N_4_ was significantly enhanced with the increase in MoO_3_ content. It is possible that the use of layered MoS_2_ as a precursor not only improves the dispersion of MoO_3_ on g-C_3_N_4_, but also enhances the interactions between them. Li et al. [166] synthesized W_18_O_49_/g-C_3_N_4_ composites by roasting a g-C_3_N_4_-impregnated ammonium tungstate solution. The loading of W_18_O_49_ greatly improves the surface area (by about five times) and exhibits excellent activity for a photocatalytic hydrogen evolution reaction, with a reaction rate of 912.3 μmol⋅g^−1^⋅h^−1^, which is 9.7 times higher than that of g-C_3_N_4_.

**Figure 16:**
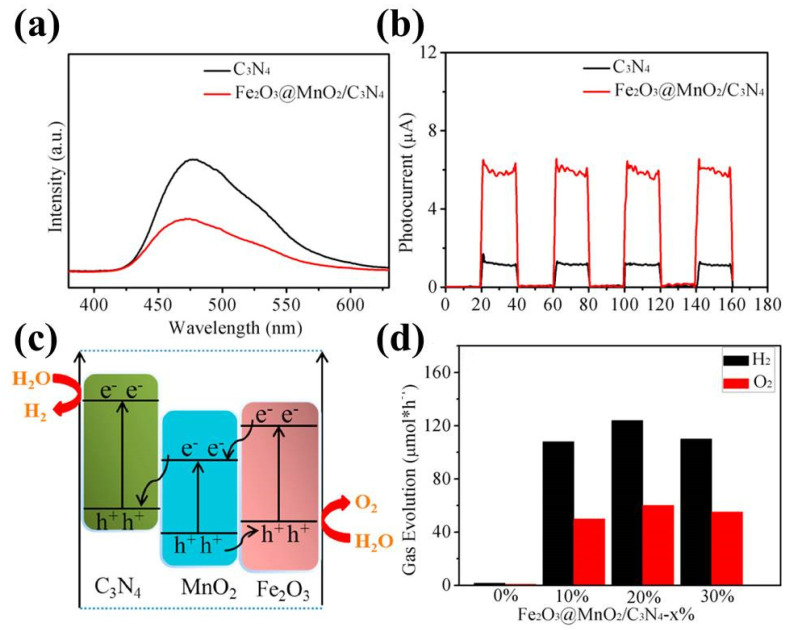
(**a**) PL spectra and (**b**) photocurrent response of C_3_N_4_ and Fe_2_O_3_@MnO_2_/C_3_N_4_ samples; (**c**) schematic diagram of electron transfer; and (**d**) activity for splitting of water into H_2_ and O_2_ for the Fe_2_O_3_@MnO_2_/C_3_N_4_ photocatalyst. Used with permission from [170]. Copyright 2020 Elsevier.

The coupling of g-C_3_N_4_ with two metal oxides could be more interesting when compared to that with single-metal oxide, as multiple heterojunctions can be established, exhibiting rich optical properties, and hence, better photocatalytic activities. This is observed in many studies [167,168,169]. For example, Wang et al. [170] found that Fe_2_O_3_@MnO_2_ core-shell g-C_3_N_4_ ternary composites can form double heterojunctions, which provide abundant channels for electrons transfer, exhibit enhanced optical properties and allow the two half-reactions (the production of hydrogen and oxygen) to occur on the opposite surfaces of the semiconductor (Figure 16a–c); this results in improved activity for both hydrogen and oxygen production, with an optimal reaction rate of 124 μmol⋅h^−1^ and 60 μmol⋅h^−1^, respectively (Figure 16d).

### 3.2. Photocatalytic Reduction of CO_2_ to Renewable Hydrocarbon Fuels

With increasing global warming, it is critical to find effective ways to deal with greenhouse gases. Carbon dioxide (CO_2_) is not only a typical greenhouse gas but also a valuable C1 resource. Hence, utilizing solar energy to reduce CO_2_ into higher-value chemicals shows great advantages in solving the problems of both global warming and energy crises. In the past few years, g-C_3_N_4_ has been employed as a photocatalyst for CO_2_ reduction owing to its high CB potential, which can activate CO_2_ by donating electrons to the unoccupied orbits of CO_2_. The photocatalytic CO_2_ reduction involves a proton-assisted multi-electron process, as shown in Equations (1)–(5) below [171]. From the viewpoint of thermodynamics, CO_2_ is gradually reduced to HCOOH, CO, HCHO, CH_3_OH and CH_4_ by receiving multiple (2, 2, 4, 6 and 8) electrons and protons, accompanying the increase in reduction potential. This means that the photocatalyst used to reduce CO_2_ should have strong redox capability in order to supply sufficient driving force for the reaction.

CO_2_ + 2H^+^ + 2e^−^ → HCOOH E^0^_redox_ = −0.61V (vs. NHE at pH 7) (1)

CO_2_ + 2H^+^ + 2e^−^ → CO + H_2_O E^0^_redox_ = −0.53V (vs. NHE at pH 7) (2)

CO_2_ + 4H^+^ + 4e^−^ → HCHO + H_2_O E^0^_redox_ = −0.48V (vs. NHE at pH 7) (3)

CO_2_ + 6H^+^ + 6e^−^ → CH_3_OH + H_2_O E^0^_redox_ = −0.38V (vs. NHE at pH 7) (4)

CO_2_ + 8H^+^ + 8e^−^ → CH_4_ + _2_H_2_O E^0^_redox_ = −0.24V (vs. NHE at pH 7) (5)

**Figure 17:**
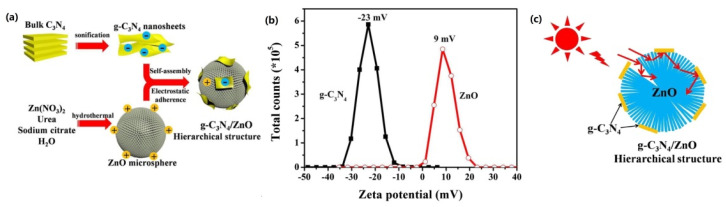
(**a**) Schematic diagram of synthesizing g-C_3_N_4_/ZnO microspheres; (**b**) zeta potential of ZnO and g-C_3_N_4_ (pH = 7); (**c**) illustration of enhanced reflections within the g-C_3_N_4_/ZnO photocatalyst. Used with permission from [173]. Copyright 2018 Elsevier.

ZnO can absorb CO_2_ and has a CB potential (E_CB_) of −0.44 eV, which is more negative than the reduction potential of CO_2_. Therefore, the combination of ZnO and g-C_3_N_4_ would benefit the CO_2_ reduction reaction. Indeed, it is found that although the deposition of ZnO has negligible effects on the light absorption capacity and surface area of g-C_3_N_4_, the ZnO/g-C_3_N_4_ composite shows better photocatalytic activity for CO_2_ reduction than individual ZnO and g-C_3_N_4_, due to the formation of heterojunctions that facilitate the separation of e^−^/h^+^ pairs [172]. The CO_2_ conversion rate obtained from ZnO/g-C_3_N_4_ reaches 45.6 μmol/g/h, which is 4.9 times and 6.4 times higher than that obtained from g-C_3_N_4_ and P25, respectively. Additionally, based on the fact that the zeta potential of ZnO is positive and that of g-C_3_N_4_ is negative, Nie et al. [173] constructed a ZnO/g-C_3_N_4_ composite using an electrostatic self-assembly method, as shown in Figure 17a,b. The combination of them induces synergistic effects that are conducive to photocatalytic reactions, in which the ZnO microsphere prevents falling g-C_3_N_4_ nano flakes from gathering, and the g-C_3_N_4_ improves light utilization efficiency through the multi-scattering effect (Figure 17c).

In addition to ZnO, many other metal oxides can couple with g-C_3_N_4_ and contribute to the CO_2_ reduction reaction. For example, Bhosale et al. [174] employed a wet chemical method to couple FeWO_4_ with g-C_3_N_4_, forming a Z-scheme g-C_3_N_4_/FeWO_4_ photocatalyst; it showed good activity for the reduction of CO_2_ to CO without any medium, with a CO production rate of 6 µmol/g/h, which is 6 and 15 times higher than that of individual g-C_3_N_4_ and FeWO_4_.

### 3.3. Photocatalytic Degradation of Pollutants

With the rapid development of the economy, various toxic pollutants emitted from industrial plants have been discharged to the environment and have seriously destroyed the ecological system. The removal of pollutants and the remediation of the environment have thus become essential topics and have attracted broad attention in recent years. Photocatalysis is a prospective technology for pollutant removal, and is able to mineralize organic pollutants into CO_2_ and H_2_O by producing oxidizing intermediates (such as •O_2_^−^, •OH and h^+^). Depending on the properties of the pollutants, three reaction types can be classified: (1) the removal of organic pollutants in aqueous solution, such as dye [166,175] and antibiotic degradation [176]; (2) the removal of heavy-metal cations in aqueous solution, such as the reduction of chromium (VI) [177]; and (3) the removal of organic or inorganic pollutants in gas phase, such as the degradation of ortho-dichlorobenzene [178], acetaldehyde [179] and nitric oxide [180].

The Fenton advanced oxidation process (with an Fe^2+^ and H_2_O_2_ system) is a traditional technology used to treat industrial wastewater, but it is limited to a narrow pH range (<3) and causes secondary pollution due to the production of iron sludge. For this reason, it is proposed that a photocatalyst should be used instead of Fe^2+^, to activate H_2_O_2_ into •OH radicals under light irradiation conditions, which can be achieved in a wide pH range without producing secondary pollutants. Hence, it is a green route to removing organic pollutants in aqueous solution and has good prospects for industrial use.

**Figure 18:**
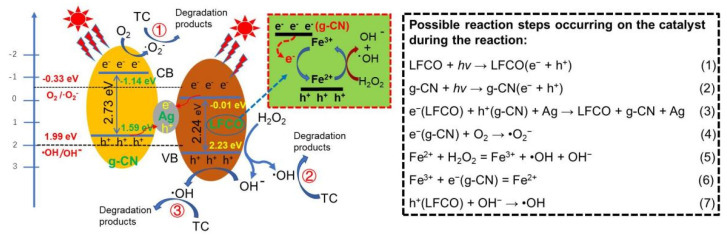
Mechanism of photo—Fenton degradation of tetracycline hydrochloride over the ternary LFCO/Ag/g-CN heterojunctions under visible-light irradiation. Used with permission from [182]. Copyright 2022 John Wiley and Sons.

In this respect, Xu et al. [181] recently reported that the LFO@CN photocatalyst is highly efficient for the oxidative degradation of RhB with H_2_O_2_ under visible-light irradiation, with 98% conversion obtained within 25 min, and the material can be recycled for four cycles with no appreciable deactivation. Moreover, when applying a ternary LaFe_0.5_Co_0.5_O_3_/Ag/g-C_3_N_4_ heterojunction that consists of a redox part LaFe_0.5_Co_0.5_O_3_ (LFCO), photo part g-C_3_N_4_ and plasmonic part (Ag), for the degradation of tetracycline hydrochloride (TC), in the presence of H_2_O_2_ and light irradiation, the system exhibits good activity due to a photo-Fenton effect induced in the reaction, as shown in Figure 18 [182]. In this system, H_2_O_2_ is first activated into •OH radicals and OH^−^ anions over the LFCO, and the OH^−^ anions subsequently react with holes (h^+^) produced at the VB band of LFCO to form more •OH radicals. Hence, H_2_O_2_ can be fully utilized to oxidize TC in the reaction. Meanwhile, the O_2_ dissolved in the solution can react with the electrons (e^−^) generated at the CB band of g-C_3_N_4_ and form •O_2_^−^, which is also a strong oxidant that is able to oxidize TC into CO_2_ and H_2_O. These results support that g-C_3_N_4_-based catalysts have good chemical stability and can be an effective substitute for Fenton catalysts in environmental purification.

In addition to the direct addition of H_2_O_2_, the photocatalytic in situ generation of H_2_O_2_ in the reaction for pollutant oxidation, which is a more promising way but a more challenging topic, is also possible. For example, Xu et al. reported that ternary g-C_3_N_4_/Co_3_O_4_/Ag_2_O heterojunctions can accelerate the mineralization of RhB due to the presence of H_2_O_2_ in situ, produced from O_2_ reduction [183]. Through studying the catalytic behavior of the composites in the electrochemical oxygen reduction reaction (ORR), they found that the average number of electrons transferred in the reaction is 2.07, which indicates that the two-electron O_2_ reduction process is the dominant step in the reaction.

The morphology of metal oxide, the interface interaction between metal oxide and g-C_3_N_4_ and the method of coupling metal oxide with g-C_3_N_4_ are also crucial factors affecting the photocatalytic performance of g-C_3_N_4_ for pollutant removal. For instance, the coupling of cubic CeO_2_ (3~10 nm) with g-C_3_N_4_ using a hydrothermal method can greatly improve the activity of g-C_3_N_4_ for methyl orange degradation, with the reaction rate reaching 1.27 min^−1^, which is 7.8 times higher than that of g-C_3_N_4_ alone (0.16 min^−1^) [184]. The hybridization of NiO with g-C_3_N_4_ causes a red shift in the UV absorption edge and boosts the ability of light response; hence, it exhibits improved activity for methylene blue degradation, which is about 2.3 times higher than that of g-C_3_N_4_ [185]. Similar phenomena are also observed for other materials, e.g., TiO_2_-In_2_O_3_@g-C_3_N_4_ [186].

**Figure 19:**
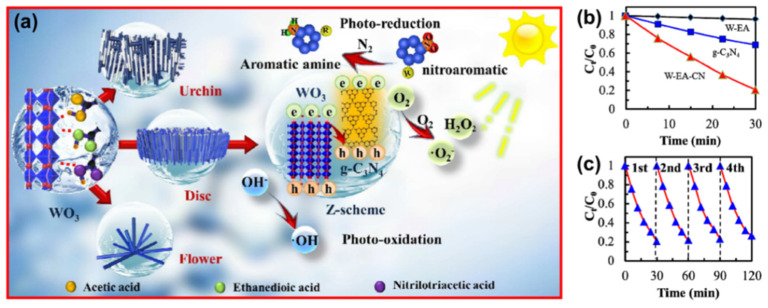
(**a**) The mechanism of organic acid inducing the growth of WO_3_ with different shapes and the photocatalytic process occurring over g-C_3_N_4_/WO_3_; (**b**) photocatalytic activity of different samples; and (**c**) recycle stability for the reduction of Cr(VI) and (m) cyclic experiments of W-EA-CN for photoreduction of Cr(VI). Used with permission from [188]. Copyright 2012 Royal Society of Chemistry.

The heavy-metal ions produced in electroplating, metallurgy, printing and dyeing, medicine and other industries cause serious damage to the ecological environment. Cr(VI) is a typical heavy metal in wastewater and its removal receives wide attention. The photocatalytic reduction of Cr(VI) to Cr(III) is an efficient way to treat Cr(VI)-containing wastewater, due to its simple process, energy savings, high efficiency and lower levels of secondary pollution [187]. It has been reported that the in situ self-assembly of g-C_3_N_4_/WO_3_ in different organic acid media can lead to various surface morphologies and catalytic activities for Cr(VI) removal, as the number of carboxyl groups in organic acid greatly affects the shape and performance of g-C_3_N_4_/WO_3_. Its synthesis in ethanedioic acid medium, which contains two carboxyl groups, yields a disc shape and has the best activity for nitroaromatic reduction (Figure 19a,b). Furthermore, the material has good stability for the reaction, with no appreciable activity loss within four cycles, as shown in Figure 19c [188].

Bi_2_WO_6_ is a promising semiconductor that can couple with g-C_3_N_4_ and form a heterojunction for the photocatalytic treatment of Cr(VI)-containing wastewater. Song et al. [189] found that a C_3_N_4_/Bi_2_WO_6_ composite prepared using a hydrothermal method exhibits a surface area up to 46.3 m^2^/g and shows a rate constant of 0.0414 min^−1^ for the photocatalytic reduction of Cr(VI), as the high surface area of the catalyst facilitates not only the reactant’s adsorption, but also the visible-light absorption.

Photocatalysis is also effective for removing gas-phase pollutants and receives great interest from scientists. It is known that air pollution is a big problem for the environment, and causes serious harm to the human body and ecological systems by forming acid rain, chemical smog, particulate matter, etc. Hence, seeking an effective and feasible technology for its removal is a challenging topic. Photocatalysis provides a way to remove air pollutants (e.g., NOx) by installing catalysts either inside the exhaust pipe or on the road surface [1]. As a typical photocatalyst, g-C_3_N_4_-based materials are also widely investigated in this aspect. Zhu et al. reported that g-C_3_N_4_ is active in NO removal via thermal catalysis, and proposed that the N atoms of g-C_3_N_4_, with a lone electron pair, serve as the active site of NO by donating electrons to weaken the N-O bond order [190]. This lays the foundation or using photocatalysis for NO removal, as electrons can be effectively excited from g-C_3_N_4_ under light irradiation.

However, it is known that the surface area of g-C_3_N_4_ prepared using the thermal condensation method is small, which grfieatly limits the light absorption capacity, the e^−^/h^+^ separation efficiency and other physicochemical properties; thus, many strategies have been adopted to overcome this problem. For example, Sano et al. [191] reported that pretreating melamine with NaOH solution before the condensation process favors the hydrolysis of unstable domains and the generation of mesopores in the structure of g-C_3_N_4_, leading to an increase in surface area from 7.7 m^2^/g to 65 m^2^/g, and the NO oxidation activity is accordingly increased 8.6 times. Duan et al. [180] found that flower-like g-C_3_N_4_ prepared using the self-assembly method can notably improve photocatalytic activity for NO oxidation compared to bulk g-C_3_N_4_, owing to the enlargement of the BET surface area, the formation of nitrogen vacancies, the condensation of π–π layer stacking, and the improvement in e^−^/h^+^ separation efficiency. The alternation of the precursor, e.g., urea [192] and guanidine hydrochloride [193] is also efficient in preparing g-C_3_N_4_ with a large surface area and improving photocatalytic performance.

### 3.4. Sterilization and Disinfection

In addition to the above applications, photocatalysis is also widely applied to inactivate pathogens in surface water owing to its broad compatibility, long durability, anti-drug resistance and thorough sterilization [194]. Bacteria, such as salmonella, staphylococcus aureus and bacillus anthracis, are commonly used as model pathogens to evaluate photocatalytic disinfection efficiency. Since the first work of Matsunaga et al. [195] on photochemical sterilization in 1985, this technique has rapidly developed and receives great interest from scientists. The principle of photocatalytic sterilization is to excite and separate the e^−^/h^+^ pairs via illumination; the photoinduced electrons and/or holes then inactivate the bacteria by directly or indirectly inflicting oxidative damage on their organs (through the formation of •O_2_^−^, •OH, etc.). Hence, the disinfection efficiency of materials closely depends on the properties that influence the generation and separation of e^−^/h^+^ pairs, e.g., the surface area, the band gap and the surface morphology, as reported for other photocatalytic processes.

**Figure 20:**
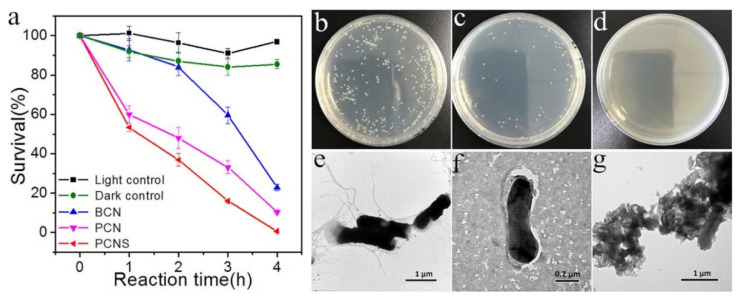
(**a**) Visible-light-driven photocatalytic disinfection performance against *E. coli* over BCN (bulk g-C_3_N _4_), PCN (porous g-C_3_N_4_) and PCNS (porous g-C_3_N_4_ nanosheets). Images of *E. coli* on solid culture medium before (**b**) and after ((**c**) 2 h; (**d**) 4 h) light irradiation on PCNS. TEM images of *E. coli* cells (**e**) before irradiation and (**f**,**g**) after disinfection for 4 h on PCNS. Used with permission from [197]. Copyright 2017 American Chemical Society.

In the case of g-C_3_N_4_, Huang et al. [196] found that mesoporous g-C_3_N_4_ synthesized using the hard template method can inactivate most of the bacteria (e.g., *E. coli* K-12) within 4 h, owing to its large surface area, which allows more active sites exposed on the surface to produce h^+^ for bacterial disinfection. To support that the inactivation of bacteria is caused by photocatalysis, Xu et al. [197] conducted a dark contrasting experiment using a porous g-C_3_N_4_ nanosheet (PCNS) as the photocatalyst and *E. coli* as the model bacteria; they found that the adsorption of *E. coli* on PCNS reaches equilibrium within 1 h and about 85.5% of *E. coli* survive after 4 h, while nearly 100% of *E. coli* are killed by PCNS within 4 h under visible-light irradiation (Figure 20a). This demonstrates that the PCNS has little toxic effect on *E. coli* and the disinfection is mainly caused by the electrons or holes induced from PCNS under light irradiation. Figure 20b–g display the morphology of *E. coli* before and after photocatalytic disinfection, observed from TEM images, showing that the bacterial cells are tightly bound to PCNS and the outer membrane is partially damaged after 4 h of irradiation.

**Figure 21:**
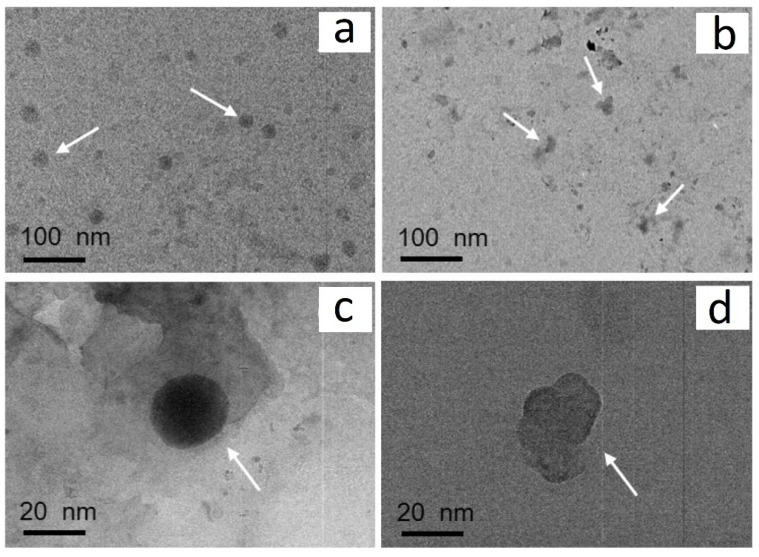
TEM images of phage MS2 before (**a**,**b**) and after (**c**,**d**) treatment with g-C_3_N_4_ for 6 h under visible-light irradiation. Used with permission from [198]. Copyright 2016 Elsevier.

In addition to bacterial infection, viral outbreaks, including SARS, bird flu, Ebola and the recent COVID-19, are also important events related to human health, and they are generally more resistant than bacteria to conventional disinfection due to their small size. Thus, the inactivation of viruses normally requires strong oxidative agents. g-C_3_N_4_-based materials have good photocatalytic reactivity to produce strong oxidative agents, e.g., •O_2_^−^ and •OH; hence, they are potential photocatalysts for virus inactivation. It has been reported that phage MS_2_ can be completely inactivated by g-C_3_N_4_ under visible-light irradiation within 360 min [198], and the main active species for the reaction are •O_2_^−^ and •OH. Figure 21 shows that the phage MS2 in contact with g-C_3_N_4_ maintains integrity before irradiation, and its structure is severely damaged after 6 h of visible-light irradiation. The loss of protein triggers the leakage and rapid destruction of internal components, and ultimately leads to the death of the virus without regrowth.

## 4. Summary and Outlook

We provide an overview of the synthesis and photocatalytic applications of g-C_3_N_4_ and its coupling with single- or multi-metal oxides. Currently, the improvement in the photocatalytic performance of g-C_3_N_4_ mainly focuses on three aspects: (1) enhancing the adsorption capacity for target reactants, (2) broadening the absorption range to visible light, and (3) improving the e^−^/h^+^ pair separation efficiency. Generally, coupling with metal oxide can almost accomplish these three aspects, by increasing the surface area, narrowing the band gap and forming heterojunctions, for example.

Coupling metal oxide semiconductors with suitable energy levels is a promising strategy to improve the activity of g-C_3_N_4_ for photocatalytic reactions, by forming type II or Z-scheme heterojunctions, which facilitate the separation, and hence, the utilization of e^−^/h^+^ pairs. Moreover, the alterable valence of transition metals enables the composites to exhibit redox properties that benefit the proceeding of reactions undergoing electron transfer steps. Hence, the coupling of metal oxide semiconductors can widen the applications of g-C_3_N_4_ and may result in synergistic effects (e.g., photo-redox) that facilitate the proceeding of complex reactions.

However, because metal oxides are easy to sinter and reduce with g-C_3_N_4_ (which can be regarded as a type of reducing agent) at high temperature, developing a suitable method to obtain desirable effects on the composites is of great importance. This can be related to (1) the surface morphology, such as surface area and pore size, which affects, for example, the ability to absorb light, the spatial separation efficiency of e^−^/h^+^ pairs and the capability to adsorb the reactant; (2) the interface interaction, which influences the mobility of electrons and/or holes between g-C_3_N_4_ and the metal oxide, either for the formation of the newly balanced band gap (for type II heterojunctions) or for the recombination of unused electrons and holes (for Z-Scheme heterojunctions); or (3) the type of heterojunction (type II or Z-Scheme) formed, which depends on the properties of metal oxide, such as the Fermi level, the band level or the band gap, and the semiconductor type (*p*- or n-type). Hence, it is essential to consider the preparation method, the properties of the metal oxide and the integrating degree between g-C_3_N_4_ and the metal oxide, to obtain the best synergistic effect and fully exhibit the photocatalytic performance of the g-C_3_N_4_/metal oxide composites.

Photocatalytic applications of g-C_3_N_4_ and g-C_3_N_4_/metal oxide composites for energy synthesis and environmental protection have been widely reported for gas-, liquid- and gas–liquid-phase reactions. Because of the redox capability of metal oxides, redox and photocatalytic processes can simultaneously occur in the reaction, and a synergistic effect may be induced between them, for instance, the photo-Fenton reaction process. This improves the reaction rate while making the reaction process complex. Hence, the reaction process deserves further exploration and investigation in order to reveal and understand the photocatalytic mechanism, such as the manners of charge transfer, the internal force-field adjustment, the electron interaction between g-C_3_N_4_ and the metal oxide, etc. The collaboration of advanced characterizations and theoretic simulation calculations would be useful in this respect and could be a development tendency in photocatalysis in future.

## Figures and Tables

**Table 1:** Surface area and band gap of g-C_3_N_4_ synthesized under different preparation conditions.

Precursor	Reaction Conditions	Band Gap [eV]	Surface Area [m^2^/g]	Ref.
Cyanamide	550 °C, 4 h, N_2_	2.62	10	[95]
Dicyandiamide	550 °C, 3 h, air	2.64	40.5	[38]
Melamine	550 °C, 3 h, air	2.66	28.2	[38]
Urea	550 °C, 3 h, air	2.72	67.1	[38]
Urea	550 °C, 2 h, air	2.76	58	[39]
Thiourea	550 °C, 2 h, air	2.58	18	[39]
3-amino-1, 2, 4-triazole	550 °C, 4 h, CO_2_	2.05	7.2	[56]
Ammonium thiocyanate	550 °C, 2 h, NH_3_	2.87	46	[55]
Guanidine hydrochlorides	550 °C, 3 h, air	2.70	16.08	[58]
Guanidine thiocyanate	550 °C, 2 h, N_2_	2.74	8	[96]
Urea Melamine	520 °C, 4 h, air	2.47	39.06	[97]
Imidazole-mixed urea	550 °C, 4 h, air	2.26	105.28	[77]
Sulfur-mixed melamine	650 °C, 2 h, N_2_	2.65	26	[98]
Melamine–cyanuric acid	550 °C, 10 h, air	2.72	142.8	[99]
H_2_SO_4_-treated melamine	600 °C, 4 h, Ar	2.69	15.6	[82]
HCl-treated melamine	550 °C, 2 h, air	2.66	24.7	[79]
HNO_3_-treated melamine	550 °C, 2 h, air	2.65	59.3	[81]

**Table 2:** Summary of recent advances in photocatalytic degradation using metal oxide/g-C_3_N_4_ composites.

Sample	Model Reaction	Reaction Activity (mol/g/min)	Refs.
CaO/g-C_3_N_4_	Degradation of MB	2.6 × 10^−5^	[112]
SrO_2_/g-C_3_N_4_	Degradation of RhB	4.5 × 10^−7^	[113]
MnO_2_/g-C_3_N_4_	Degradation of MO	9.4 × 10^−7^	[108]
ZnO/g-C_3_N_4_	Degradation of MO	3.3 × 10^−7^	[114]
MoO_3_/g-C_3_N_4_	Degradation of MB	9.7 × 10^−7^	[115]
AgO/g-C_3_N_4_	Degradation of RhB	5.2 × 10^−6^	[116]
CdO/g-C_3_N_4_	Degradation of RhB	1.1 × 10^−7^	[117]
In_2_O_3_/g-C_3_N_4_	Degradation of RhB	5.2 × 10^−10^	[118]
SnO_2_/g-C_3_N_4_	Degradation of MO	7.9 × 10^−8^	[119]
TiO_2_/g-C_3_N_4_	Degradation of RhB	9.3 × 10^−9^	[120]
Bi_2_O_3_/g-C_3_N_4_	Degradation of Amido black 10B dye	3.3 × 10^−7^	[121]
Nb_2_O_5_/g-C_3_N_4_	Degradation of tetracycline	5.3 × 10^−7^	[122]
CeO_2_/g-C_3_N_4_	Degradation of Norfloxacin	4.6 × 10^−7^	[123]
ZnO/NiFe_2_O_4_/g-C_3_N_4_	Degradation of LVX	1.7 × 10^−7^	[124]
TiO_2_/ZnO/g-C_3_N_4_	Degradation of MB	4.9 × 10^−7^	[125]
Fe_3_O_4_/BiOBr/g-C_3_N_4_	Degradation of TC	8.8 × 10^−7^	[126]
ZnO/CuO/g-C_3_N_4_	Degradation of MB	6.1 × 10^−6^	[127]
WO_3_/Fe_3_O_4_/g-C_3_N_4_	Degradation of diazinon	6.5 × 10^−7^	[128]
Ni_3_(VO_4_)_2_/ZnCr_2_O_4_/g-C_3_N_4_	Degradation of p-CP	4.8 × 10^−4^	[129]

MO: methyl orange; MB: methylene blue; RhB: rhodamine B; LVX: levofloxacin; TC: tetracycline; p-CP: p-chlorophenol.

**Table 3:** Summary of recent advances in photocatalytic hydrogen evolution using metal oxide/g-C_3_N_4_ composites.

Sample	Reaction Activity (mol/g/min)	Refs.
Al_2_O_3_/g-C_3_N_4_	Al_2_O_3_/g-C_3_N_4_: 8.7 × 10^−6^ g-C_3_N_4_: 3.5 × 10^−6^	[130]
CoO/g-C_3_N_4_	CoO/g-C_3_N_4_: 8.4 × 10^−7^ CoO: 4.9 × 10^−8^ g-C_3_N_4_: 8.3 × 10^−8^	[131]
NiO/g-C_3_N_4_	NiO/g-C_3_N_4_: 2.5 × 10^−7^ g-C_3_N_4_: 2.7 × 10^−9^	[132]
Cu_2_O/g-C_3_N_4_	Cu_2_O/g-C_3_N_4_: 4.0 × 10^−6^ g-C_3_N_4_: 2.4 × 10^−6^	[133]
MgO/g-C_3_N_4_	MgO/g-C_3_N_4_: 5.0 × 10^−6^ g-C_3_N_4_: 9.7 × 10^−7^	[134]
**FEO_X_/G-C_3_N_4_**	FeO_x_/g-C_3_N_4_: 1.8 × 10^−5^ g-C_3_N_4_: 4.3 × 10^−6^	[110]
